# Hmga2 is dispensable for pancreatic cancer development, metastasis, and therapy resistance

**DOI:** 10.1038/s41598-018-32159-x

**Published:** 2018-09-18

**Authors:** Shin-Heng Chiou, Madeleine Dorsch, Eva Kusch, Santiago Naranjo, Margaret M. Kozak, Albert C. Koong, Monte M. Winslow, Barbara M. Grüner

**Affiliations:** 1 Department of Genetics, Stanford University School of Medicine, Stanford, CA USA; 2 Department of Medical Oncology, West German Cancer Center, University Hospital Essen, University Duisburg-Essen, Essen, Germany; 3 Department of Radiation Oncology, Stanford University School of Medicine, Stanford, CA USA; 4 Present Address: Department of Radiation Oncology, MD Anderson Cancer Center, The University of Texas, Houston, TX USA; 5 Department of Pathology, Stanford University School of Medicine, Stanford, CA USA; 6 Stanford Cancer Institute, Stanford University School of Medicine, Stanford, CA USA; 7 German Cancer Consortium (DKTK) partner site Essen/Düsseldorf, Essen, Germany

## Abstract

Expression of the chromatin-associated protein HMGA2 correlates with progression, metastasis and therapy resistance in pancreatic ductal adenocarcinoma (PDAC). Hmga2 has also been identified as a marker of a transient subpopulation of PDAC cells that has increased metastatic ability. Here, we characterize the requirement for Hmga2 during growth, dissemination, and metastasis of PDAC *in vivo* using conditional inactivation of Hmga2 in well-established autochthonous mouse models of PDAC. Overall survival, primary tumour burden, presence of disseminated tumour cells in the peritoneal cavity or circulating tumour cells in the blood, and presence and number of metastases were not significantly different between mice with Hmga2-wildtype or Hmga2-deficient tumours. Treatment of mice with Hmga2-wildtype and Hmga2-deficient tumours with gemcitabine did not uncover a significant impact of Hmga2-deficiency on gemcitabine sensitivity. Hmga1 and Hmga2 overlap in their expression in both human and murine PDAC, however knockdown of *Hmga1* in Hmga2-deficient cancer cells also did not decrease metastatic ability. Thus, Hmga2 remains a prognostic marker which identifies a metastatic cancer cell state in primary PDAC, however Hmga2 has limited if any direct functional impact on PDAC progression and therapy resistance.

## Introduction

Pancreatic ductal adenocarcinoma (PDAC) is among the most fatal of all malignancies^1^. Due to its high rate of metastatic spread and poor response to therapy the five-year survival rate for PDAC patients remains below ten percent and PDAC is projected to be the second most common cause of cancer deaths by 2030^2^. While PDAC is well characterized genomically,^3,4^ the mechanisms that enable cancer cells to leave their primary site and form distant metastases remain largely unknown^5–10^. The most common driver mutations in human PDAC are oncogenic mutations in KRAS and loss of the tumour suppressors CDKN2A, SMAD4, and TP53. These alterations are present in both primary tumours and metastases and additional genomic alterations specific to metastases have not been identified^6,11^. Several recent studies have suggested that PDAC metastasis is driven by epigenetic alterations, metabolic changes, and microenvironment-induced gene expression alterations^10,12–15^. Due to its highly metastatic nature, most PDAC patients are diagnosed at an advanced disease stage with local or distant metastasis and are therefore not candidates for potentially curative surgery. Thus, the majority of patients are treated with chemotherapy. However, chemotherapeutic regimens and targeted therapies are largely ineffective, and tumours that do respond rapidly develop resistance^12,15–17^.

Genetically engineered mouse models of PDAC recapitulate many aspects of the human disease. Pancreas-specific expression of oncogenic Kras^G12D^ and *Cre/loxP*-based inactivation of the tumour suppressors that are frequently inactivated in human PDAC results in the development of murine PDAC that is histologically and molecularly similar to the human disease^18,19^. These models recapitulate the entire course of the disease, from the development of preneoplastic lesions to invasive carcinomas and the development of widespread metastatic disease^19–22^. Importantly, these models also allow the impact of additional genetic alterations to be investigated during the initiation and progression of pancreatic cancer, as well as response to therapy *in vivo*^23–25^.

HMGA2 (high mobility group A2) is a chromatin-associated DNA-binding protein that is expressed in embryonic stem cells and during development, but is absent from most adult somatic cells^26,27^. Interestingly, HMGA2 is re-expressed in many human malignancies, including PDAC, where high expression correlates with lymph node metastasis, increased tumour grade, and reduced patient survival^28–34^. HMGA2 has been shown to interact with histones and alter DNA topology through binding to the minor groove of DNA^26^. Due to its high expression in multiple cancer types at advanced stages as well as in metastases, it has been suggested that HMGA2 functions to drive metastatic ability^27,28,30,34–39^. Recently, using genetically engineered mouse models, we defined a transient, Hmga2-positive subpopulation of PDAC cells that has increased metastatic ability^13^. However, it remains unclear whether Hmga2 is a functionally important driver of the pro-metastatic properties of these cells or only a marker of this state.

In this study, we directly investigated the importance of Hmga2 during PDAC development, metastasis, and therapy response *in vivo*. We conditionally inactivated Hmga2 in well-established autochthonous PDAC models and found no effect of Hmga2 inactivation on tumour initiation, progression, or metastasis. The absence of Hmga2 also did not dramatically alter the response of autochthonous PDAC to gemcitabine treatment.

## Results

### Hmga2 deficiency does not impact the survival of PDAC bearing mice

**Figure 1:**
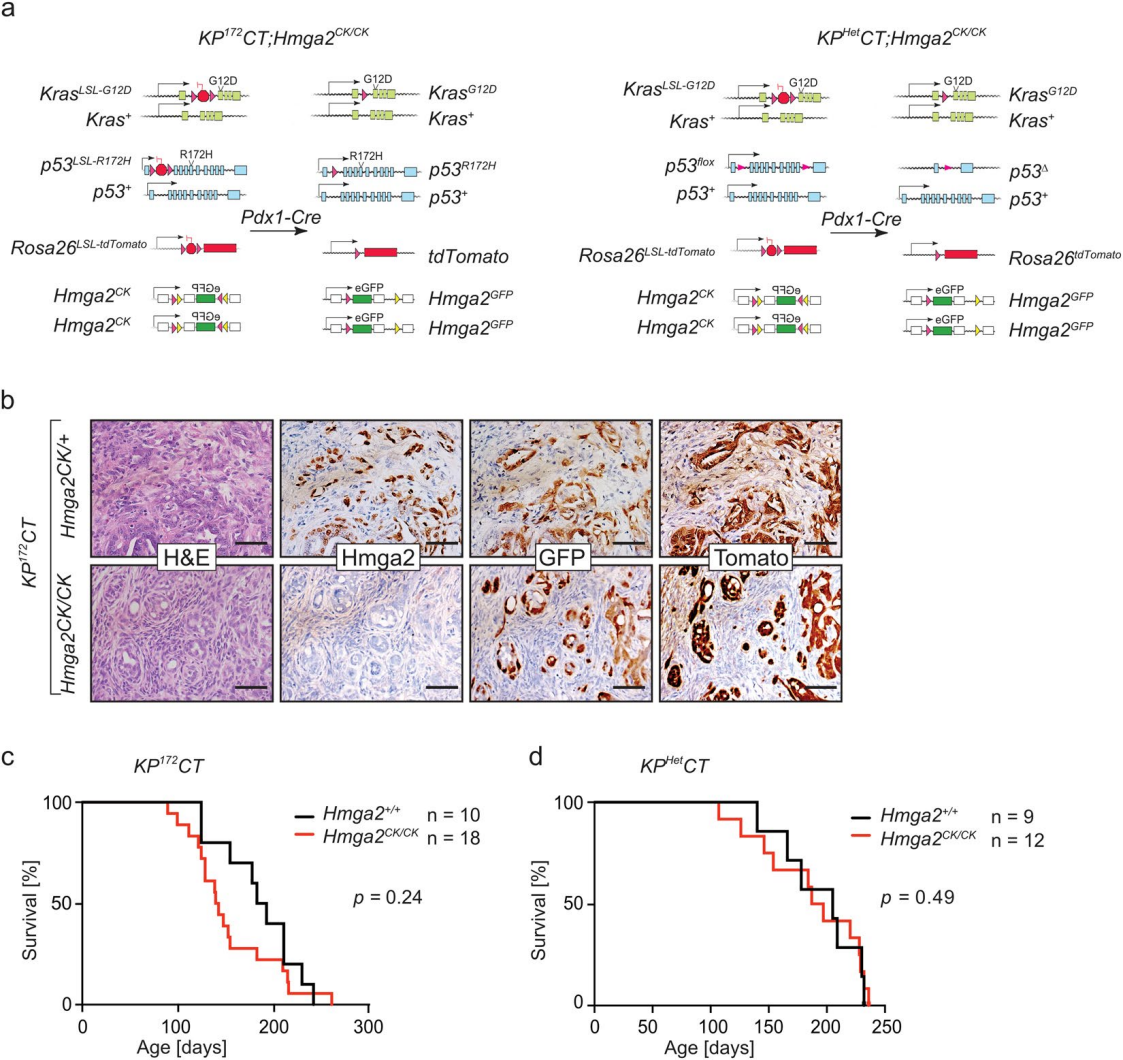
Absence of Hmga2 does not alter overall survival in autochthonous mouse models of PDAC. (**a**) Alleles in the *KP*^172^*CT* (*Kras*^*LSL-G12D/*+^*;p53*^*LSL-R*172*H/*+^*;R26*^*LSL-Tom/*+^*;Pdx1-Cre*) and the *KP*^*Het*^*CT* (*Kras*^*LSL-G12D/*+^*;p53*^*flox/*+^*;R26*^*LSL-Tom/*+^*;Pdx1-Cre*) models before and after allele recombination. (**b**) Hmga2 expression in pancreatic tumors in *KP*^172^*CT;Hmga2*^*CK/*+^ mice overlaps with GFP in Tomato positive cancer cells. Hmga2 is absent in *KP*^172^*CT*;*Hmga2*^*CK/CK*^ tumours. Representative of >10 tumors. Scale bars = 50 µm. (**c,d**) Kaplan-Meier survival curves of *KP*^172^*CT* (**c**) and *KP*^*Het*^*CT* (**d**) mice shows no significant difference with (*Hmga2*^*CK/CK*^) or without (*Hmga2*^+/+^) Hmga2 inactivation.

To investigate the role of Hmga2 in PDAC, we first confirmed Hmga2 expression in the carcinoma stage of pancreatic cancer tissue isolated from the well-established autochthonous *Kras*^*LSL-G12D/*+^*;p53*^*LSL-R*172*H/*+^*;Rosa26*^*LSL-tdTomato/*+^*;Pdx1-Cre* (*KP*^172^*CT*) mouse model of PDAC (Fig. 1a, Supplementary Fig. S1a). Recently, we uncovered Hmga2 as a marker of a transient subpopulation of pancreatic cancer cells with increased metastatic ability in *KP*^172^*CT* mice^13^. To mark Hmga2^positive^ cells within autochthonous PDAC, we utilized a conditional *Hmga2* allele that converts from the unrecombined *Hmga2*^*CK*^ conformation to a null-allele/GFP-knock-in reporter (*Hmga2*^*GFP*^) upon Cre-mediated inversion of a splice-acceptor-GFP-splice-donor cassette^13,40^.

To initially investigate whether pancreas-specific inactivation of Hmga2 would grossly alter pancreatic development and size, we generated *Hmga2*^*CK/CK*^*;Pdx1-Cre* mice. These mice were born at expected Mendelian ratios and developed normally. Pancreata from 4-week and 11-week-old mice showed no signs of malformation of the exocrine or endocrine compartments, as assessed both by organ weight and histology (Supplementary Fig. S1b,c).

To investigate the role of Hmga2 during pancreatic tumourigenesis, we incorporated the *Hmga2*^*CK*^ allele and a *Rosa26*^*LSL-tdTomato/*+^ reporter allele into the *Kras*^*LSL-G12D/*+^*;p53*^*LSL-R*172*H/*+^*;Pdx1-Cre* (*KP*^172^*C*) background (Fig. 1a). We generated *KP*^172^*CT;Hmga2*^*CK/*+^ and *KP*^172^*CT;Hmga2*^*CK/CK*^ tumour-bearing mice. Immunohistochemistry for Hmga2, tdTomato, and the GFP fusion protein confirmed that Hmga2 expression in neoplastic cells in *KP*^172^*CT;Hmga2*^*CK/*+^ mice overlaps with GFP expression and that Hmga2 protein is absent from all tumours in *KP*^172^*CT;Hmga2*^*CK/CK*^ mice (Fig. 1b). To analyse whether Hmga2 deficiency impacts the development and progression of PDAC, we generated cohorts of *KP*^172^*CT;Hmga2*^*CK/CK*^ and *KP*^172^*CT;Hmga2*^+/+^ mice. Gross cancer-associated phenotypes were comparable, with mice showing similar cachexic symptoms as well as the development of ascites and jaundice in some mice (data not shown). Kaplan-Meier analysis showed no significant survival difference between *KP*^172^*CT;Hmga2*^*CK/CK*^ and *KP*^172^*CT;Hmga2*^+/+^ mice. There was even a trend towards slightly shorter survival of *KP*^172^*CT;Hmga2*^*CK/CK*^ mice (Fig. 1c).

To further investigate the impact of Hmga2 deficiency on PDAC growth, we employed a second mouse model of PDAC, where instead of expression of a dominant negative *p53* allele (*p53*^*LSL-R*172*H*^) we incorporated a *p53*^*floxed*^ allele to enable conditional heterozygous deletion of *p53* (*p53*^*flox/*+^, Fig. 1a right panel). We generated *Kras*^*LSL-G12D/*+^*;p53*^*flox/*+^*;Rosa26*^*LSL-tdTomato/*+^;*Pdx1-Cre;Hmga2*^+/+^ (*KP*^*Het*^*CT;Hmga2*^+/+^) and *KP*^*Het*^*CT;Hmga2*^*CK/CK*^ mice and assessed overall survival. Consistent with our data from the *KP*^172^*CT* model, Hmga2-deficiency did not significantly alter overall survival and gross disease-associated phenotypes in both groups were comparable (Fig. 1d and data not shown). Collectively, these data suggest that Hmga2 inactivation has limited, if any, effect on overall pancreatic tumour growth.

### Hmga2 deficiency does not change PDAC phenotypes

**Figure 2:**
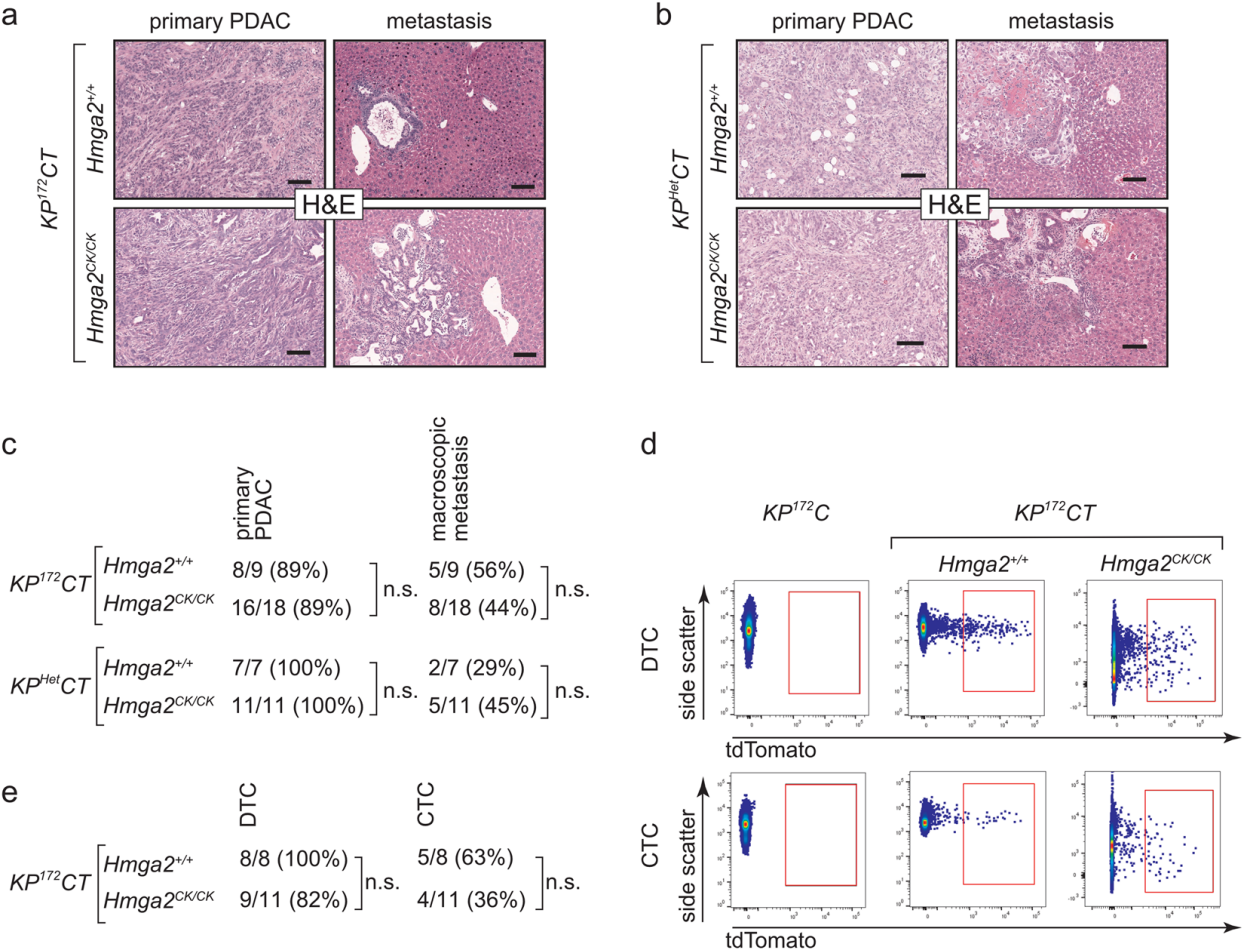
Hmga2 deficiency does not alter the phenotype of pancreatic cancer in multiple autochthonous mouse models. (**a,b**) Representative histology of primary PDAC tumours and metastases from *KP*^*172*^*CT* (**a**) and *KP*^*Het*^*CT* (**b**) mice with (*Hmga2*^+/+^) or without (*Hmga2*^*CK/CK*^) Hmga2. Scale bars = 100 µm. (**c**) Incidence of primary PDAC and metastases in *KP*^*172*^*CT* and *KP*^*Het*^*CT* mice with (*Hmga2*^+/+^) or without (*Hmga2*^*CK/CK*^) Hmga2. All comparisons showed no significant differences. (**d,e**) Incidence of circulating tumour cells (CTC) and disseminated tumour cells (DTC) is comparable in *KP*^*172*^*CT;Hmga2*^+/+^ and *KP*^*172*^*CT;Hmga2*^*CK/CK*^ mice (**e**) as detected by FACS (**d**) for tdTomato^pos^, lineage^neg^, DAPI^neg^ cancer cells in blood (CTC) and pleural fluid (DTC). No significant differences in CTC number were observed.

To investigate the impact of Hmga2 deficiency on pancreatic cancer phenotypes in *KP*^172^*CT* and *KP*^*Het*^*CT* mice, we assessed tumour histology. At the time of analysis, most *KP*^172^*CT;Hmga2*^+/+^ and *KP*^172^*CT;Hmga2*^*CK/CK*^ animals had developed pancreatic adenocarcinoma (Fig. 2a,c). In these mice, tumours were of similar size and were characterized by the presence of mPanINs and glandular structures accompanied by an extensive desmoplastic reaction (data not shown). Regardless of genotype, tumours had both well- and poorly-differentiated areas which is typical for tumours in this model^18^. Detailed histological analyses did not uncover any differences in the histological features of mice with *Hmga2*-deficient or *Hmga2*-wildtype tumours. Similarly, *KP*^*Het*^*CT;Hmga2*^+/+^ and *KP*^*Het*^*CT;Hmga2*^*CK/CK*^ mice developed pancreatic cancer with full penetrance and comparable histology (Fig. 2b,c). Thus, Hmga2-deficiency does not affect the histological phenotype of mouse pancreatic cancer.

To assess the effect of Hmga2-deficiency on cancer cell dissemination and metastasis, we quantified the development of -metastases by fluorescence-stereomicroscopy. Distant metastases were present in the liver, peritoneal cavity, and lung in about 30–50% of mice. Hmga2-deficient and Hmga2-wildtype metastases in the *KP*^172^*CT* and *KP*^*Het*^*CT* models were histologically indistinguishable (Fig. 2a–c).

We also utilized the bright fluorescent marking conferred by the *Rosa26*^*tdTomato/*+^ allele to assess the presence of disseminated tumour cells (DTCs) in the peritoneal fluid and circulating tumour cells (CTCs) in the blood by flow cytometry for tdTomato^+^ cancer cells (Fig. 2d). DTCs were present in the peritoneal cavity of almost all *KP*^172^*CT;Hmga2*^+/+^ and *KP*^172^*CT;Hmga2*^*CK/CK*^ mice and CTCs were present in five out of eight *KP*^172^*CT;Hmga2*^+/+^ and four out of eleven *KP*^172^*CT;Hmga2*^*CK/CK*^ mice. The number of CTCs detectable in mice was variable, but was not significantly different between *KP*^172^*CT;Hmga2*^+/+^ and *KP*^172^*CT;Hmga2*^*CK/CK*^ mice (Fig. 2e and Supplemental Fig. S2). Collectively, these data suggest that Hmga2 is not required for dissemination and metastasis of pancreatic cancer.

### Hmga2 deficiency does not alter PDAC sensitivity to gemcitabine

**Figure 3:**
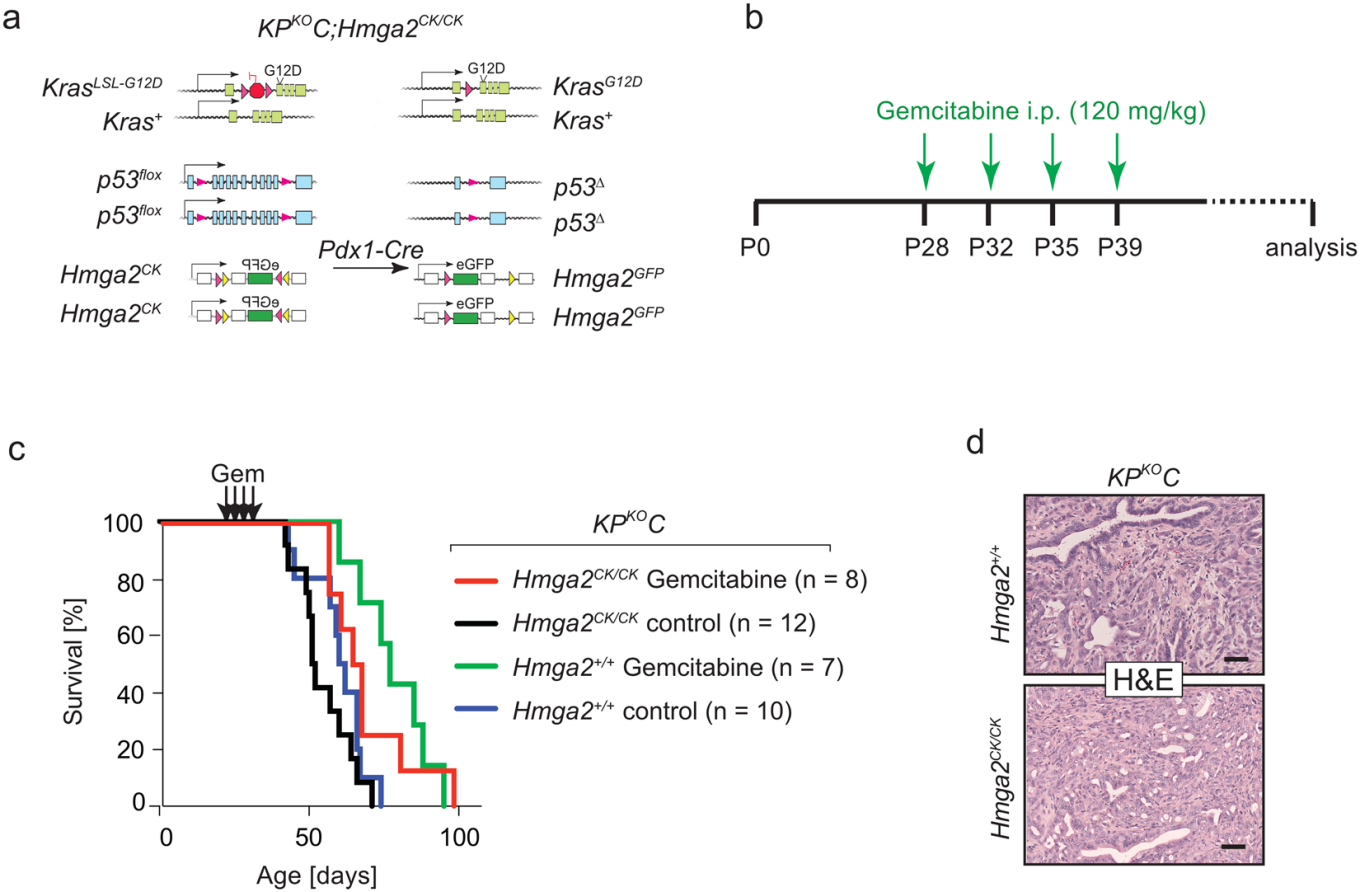
Hmga2 deficiency does not dramatically alter PDAC sensitivity to gemcitabine. (**a–c**) *KP*^*KO*^*C;Hmga2*^+/+^ and *KP*^*KO*^*C;Hmga2*^*CK/CK*^ mice (**a**) were treated with four intraperitoneal gemcitabine injections at 28, 32, 35, and 39 days of age (**b**) and compared to their respective vehicle-treated control littermates. Gemcitabine treatment slightly increased median survival in both genotypes (65.5 days in *KP*^*KO*^*C;Hmga2*^*CK/CK*^ and 77 days in *KP*^*KO*^*C;Hmga2*^+/+^ mice) compared to untreated controls (51.5 days in *KP*^*KO*^*C;Hmga2*^*CK/CK*^ and 61 days in *KP*^*KO*^*C;Hmga2*^+/+^ mice), but no significant survival difference was observed between gemcitabine -treated *KP*^*KO*^*C;Hmga2*^*CK/CK*^ and *KP*^*KO*^*C;Hmga2*^+/+^ mice (*p* = 0.47) (**c**). (**d**) Representative histology of tumours in *KP*^*KO*^*C;Hmga2*^+/+^ and *KP*^*KO*^*C;Hmga2*^*CK/CK*^ mice treated with gemcitabine. Across all mice, no consistent changes in histology were observed. Scale bars = 50 µm.

The nucleoside analogue gemcitabine is one of the standard agents of care for PDAC patients^12^. In addition to its association with more advanced and metastatic human PDAC, HMGA2 has also been suggested to promote gemcitabine resistance of PDAC^41,42^. To investigate whether Hmga2 deficiency would increase PDAC sensitivity to gemcitabine *in vivo*, we assessed the impact of Hmga2 deficiency on overall survival with and without gemcitabine treatment. In the *Kras*^*LSL-G12D/*+^*;p53*^*flox/flox*^*;Pdx1-Cre* (*KP*^*KO*^*C*) model, pancreatic cancer develops rapidly and advanced disease is already present 4 weeks after birth. *KP*^*KO*^*C* mice typically succumb to their very aggressive disease between 50 and 60 days of age^43^. To determine whether Hmga2 deficiency leads to increased sensitivity to gemcitabine, we generated *KP*^*KO*^*C;Hmga2*^+/+^ and *KP*^*KO*^*C;Hmga2*^*CK/CK*^ mice (Fig. 3a). These mice were either treated with four intraperitoneal injections of gemcitabine (120 mg/kg) or vehicle control on days 28, 32, 35 and 39 of age (Fig. 3b). We monitored overall survival as a metric of tumour response. Consistent with published reports, gemcitabine provided a small survival benefit to *KP*^*KO*^*C;Hmga2*^+/+^ mice (median survival of 61 days in control treated mice versus 77 days in gemcitabine treated mice)^24,44^. However, the increase in survival was equally small in *KP*^*KO*^*C;Hmga2*^*CK/CK*^ mice (16-day increase in survival in *KP*^*KO*^*C;Hmga2*^+/+^ mice treated with gemcitabine versus a 14-day increase in survival in *KP*^*KO*^*C;Hmga2*^*CK/CK*^ mice treated with gemcitabine) and no significant difference in survival was detectable between gemcitabine treated *KP*^*KO*^*C;Hmga2*^+/+^ and gemcitabine treated *KP*^*KO*^*C;Hmga2*^*CK/CK*^ mice (p = 0.515). This suggests that the absence of Hmga2 does not increase PDAC sensitivity to this standard chemotherapeutic agent. Furthermore, histological analysis of the tumours from gemcitabine treated *KP*^*KO*^*C;Hmga2*^+/+^ and *KP*^*KO*^*C;Hmga2*^*CK/CK*^ mice uncovered comparable histology, with complete destruction of normal pancreatic architecture and development of undifferentiated and anaplastic PDAC (Fig. 3c,d). Thus, Hmga2 deficiency did not lead to gemcitabine sensitivity *in vivo*.

### Hmga1 expression overlaps with Hmga2 expression, but Hmga1 does not compensate for Hmga2 deficiency

**Figure 4:**
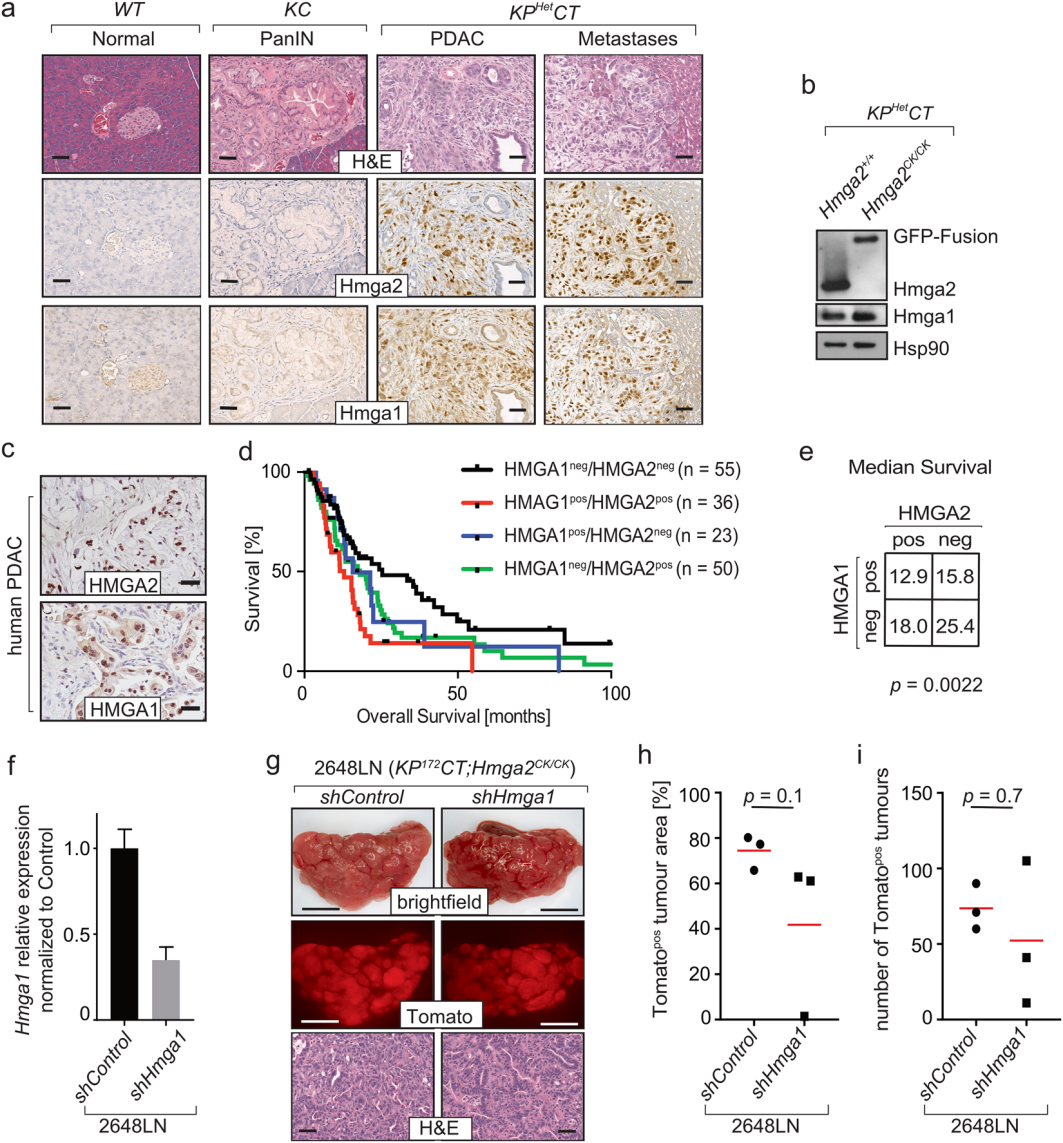
Hmga1 and Hmga2 are co-expressed in mouse and human pancreatic cancer. (**a**) Immunohistochemistry for Hmga1 and Hmga2 in wild type (*WT*), *Kras*^*LSL-G12D/*+^*;Pdx1-Cre (KC)* and *Kras*^*LSL-G12D/*+^*;p53*^*flox/*+^*;R26*^*LSL-Tom/*+^*;Pdx1-Cre* (*KP*^*Het*^*CT*) mice shows co-expression of Hmga1 and Hmga2 in PDAC and metastases but not in preneoplastic lesions or healthy pancreata. Scale bars = 50 µm. (**b**) Western Blot on sorted cancer cells from *KP*^*Het*^*CT;Hmga2*^+/+^ and *KP*^*Het*^*CT;Hmga2*^*CK/CK*^ mice documents the expression of the GFP-fusion reporter gene and the absence of Hmga2 in cells from *KP*^*Het*^*CT;Hmga2*^*CK/CK*^ mice. Hmga1 expression is not affected. Corresponding full length images and details regarding probing of the membranes are available in Supplemental Fig. S4c. (**c**) Tissue microarrays from human pancreatic cancer patients were stained for HMGA1 and HMGA2. Scale bars = 50 µm. (**d**) Kaplan-Meier survival analysis for patients with HMGA1 and HMGA2 positive or negative tumours. (**e**) Median survival in months of PDAC patients with expression of HMGA1 and HMGA2 shows that patients with HMGA1^negative^/HMGA2^negative^ tumours have significantly (*p* = 0.0022) better overall survival than patients who have tumors that express of either HMGA1 or HMGA2 or both. (**f–i**) Knockdown of *Hmga1* in a cell line (2648LN) isolated from a lymph node metastasis from a *KP*^*172*^*CT;Hmga2*^*CK/CK*^ mouse has no significant impact on their metastatic potential upon intravenous injection into recipient mice. (**f**) qPCR was used to assess knockdown; mean +/− SEM of triplicate wells is shown. (**g**) Representative brightfield and fluorescent images of one lung lobe and corresponding histology. Scale bars in the upper and middle panels = 4 mm; scale bars in the lower panels = 50 µm. (**h,i**) Quantification of %-Tomato^positive^ area (**h**) and number of tdTomato^positive^ tumours in the lung (**i**), n = 3 mice per group; the red line indicates the mean.

HMGA2 belongs to the family of HMGA proteins that consists of two family members, HMGA1 and HMGA2^26^. Both proteins are considered architectural transcription factors that regulate transcription indirectly by altering DNA conformation. In normal adult cells, both family members are almost undetectable though they are abundant during embryonic development. Both family members have been shown to regulate cell signalling, proliferation, and differentiation with at least partially overlapping functions^26,27,31,45^. The expression of both HMGA1 and HMGA2 has been shown to correlate with advanced tumour stages and metastasis in PDAC patients^32^. To investigate whether Hmga1 expression overlaps with Hmga2 expression, we performed immunohistochemistry and western blot analysis on murine and human PDAC tissue samples. In tumours from *KP*^172^*CT;Hmga2*^*CK/*+^ mice, Hmga1 and Hmga2 were expressed in primary tumour areas as well as in metastases in liver and lung (Supplemental Fig. 3a). In PDAC from *KP*^*Het*^*CT;Hmga2*^+/+^ mice the expression of Hmga1 and Hmga2 generally overlapped, with Hmga1 also being highly expressed in advanced primary tumours as well as in metastases (Fig. 4a). In PDAC from *KP*^*Het*^*CT;Hmga2*^*CK/CK*^ mice, Hmga1 was still expressed at similar levels. Western blot analysis for Hmga1 and Hmga2 on sorted (lineage^negative^, DAPI^negative^, Tomato^positive^) cancer cells isolated from pancreatic tumours from *KP*^*Het*^*CT;Hmga2*^+/+^ and *KP*^*Het*^*CT;Hmga2*^*CK/CK*^ mice confirmed the absence of Hmga2, the presence of the GFP fusion protein in Hmga2-deficient tumours, and unaltered Hmga1 expression (Fig. 4b). Similarly, HMGA1 and HMGA2 proteins are often co-expressed in human PDAC (Fig. 4c). In TCGA PDAC dataset, *HMGA1* and *HMGA2* expression was also moderately correlated (0.414 Pearson correlation coefficient or 0.581 Spearman correlation coefficient, respectively). Correlation of overall survival with HMGA1 and HMGA2 protein expression showed that PDAC patients with tumours that were both HMGA1^negative^ and HMGA2^negative^ had better overall survival than patients whose tumours expressed HMGA1 and/or HMGA2 (Fig. 4d,e; *p* = 0.0022).

To test whether Hmga1 compensates for Hmga2 in PDAC metastasis, we generated PDAC cell lines from tumours from *KP*^172^*CT;Hmga2*^+/+^ and *KP*^172^*CT;Hmga2*^*CK/CK*^ mice (n = 4 each). We performed qPCR analysis for Hmga1 and Hmga2 expression. *Hmga1* expression was not affected by *Hmga2*-deficiency (Supplemental Fig. S3c,d). To investigate if Hmga1 could functionally compensate for Hmga2, we stably knocked down *Hmga1* in a cancer cell line generated from a *KP*^172^*CT;Hmga2*^*CK/CK*^ lymph node metastasis. Knockdown at the RNA level was confirmed by qPCR (Fig. 4f). However, no significant difference in metastatic ability between control and *Hmga1* knockdown cells was observed upon intravenous injection into immunocompetent recipient mice (Fig. 4g,h,i). Additionally, we stably knocked down *Hmga1* or *Hmga2* in a cell line generated from a liver metastasis in a *KP*^172^*CT;Hmga2*^+/+^ mouse (Supplemental Fig. S4a). Intravenous transplantation of *Hmga1* knockdown, *Hmga2* knockdown, or control cells into immunocompetent recipient mice to seed metastases into the lungs showed no difference in the metastatic ability of control cells and cells deficient for either *Hmga1* or *Hmga2* (Supplemental Fig. S4b).

## Discussion

To investigate the function of the chromatin-associated protein Hmga2 during pancreatic cancer growth, metastasis, and treatment response, we took a classical genetic approach of specifically inactivating Hmga2 protein expression in the well-established *KP*^172^*CT, KP*^*Het*^*CT*, and *KP*^*KO*^*C* mouse models of PDAC. To our surprise, Hmga2-deficient tumours formed in the pancreata of *KP*^172^*CT;Hmga2*^*CK/CK*^*, KP*^*Het*^*CT;Hmga2*^*CK/CK*^, and *KP*^*KO*^*C*;*Hmga2*^*CK/CK*^ mice with similar rate and morphology and within a comparable time course as in control *KP*^172^*CT, KP*^*Het*^*CT* and *KP*^*KO*^*C* mice (Fig. 2). By all metrics, from overall survival to histology of primary tumours and metastases to the presence and number of CTCs and DTCs, Hmga2-deficiency did not perturb PDAC development in any significant way.

Numerous *in vitro* studies across several different human cancer types have suggested a role for HMGA2 in controlling phenotypes associated with malignant transformation and metastasis^32,36,42,46,47^. However, very few studies have investigated Hmga2 function in autochthonous cancer models *in vivo*. In a mouse model of Wnt1-driven colorectal cancer, tumour formation was reduced in Hmga2^−/−^ mice, suggesting a role for Hmga2 in cancer development^48^. However, Hmga2^−/−^ mice have severe dwarfism^49^, therefore the direct importance of Hmga2 in colorectal cancer cells *in vivo* remains unclear. Furthermore, in squamous skin cancer mouse models, Hmga2 was found to be dispensable for tumour initiation and progression^50^. While we recently showed that Hmga2 is a marker of a transient metastatic subpopulation in PDAC *in vivo*^13^, our current study shows that neither deletion of Hmga2 in autochthonous mouse models of PDAC, nor knockdown of *Hmga2* in an aggressive pancreatic cancer cell line impacted pancreatic tumour initiation, progression, or metastatic ability. In the genetically engineered mouse models that we used, Hmga2 deletion occurs concomitantly with expression of Kras^G12D^ and inactivation of p53 during tumour initiation, and therefore tumours develop in the complete absence of Hmga2. This could lead to an alternative, Hmga2-independent mechanism for PDAC growth and progression. Second hit mouse models of PDAC could provide additional information on whether acute Hmga2 inactivation in PDAC impacts growth, progression, or therapy response.

In contrast to human non-small cell lung cancer cell lines^35^, cell lines derived from the Kras^G12D^-driven p53-deficient mouse lung adenocarcinoma model^38^, and the mouse breast cancer cell line 4T1^48^, *Hmga2* knockdown in murine PDAC cells did not reduce metastatic ability *in vivo*. This is particularly interesting as PDAC is among the most genetically stable cancer entities and its progression is based on epigenetic changes rather than acquisition of additional DNA mutations^11,14^, suggesting an even greater importance of epigenetic reprogramming of malignant cells^39,51^.

HMGA2 has been previously implicated in resistance to gemcitabine in human pancreatic cancer cell lines *in vitro*, where *HMGA2* knockdown sensitized PDAC cells to gemcitabine treatment^42^. HMGA2 mediated gemcitabine resistance in three-dimensional collagen cultures of PDAC cell lines via increased histone acetylation^41^. To test whether Hmga2 mediates gemcitabine resistance *in vivo*, we utilized a well-established clinically relevant treatment model^44,52,53^. Neither overall survival nor histology of the tumours in mice treated with gemcitabine were significantly affected by Hmga2-deficiency. While these models have limited resolution to detect modest effects, our results suggest that Hmga2 has limited if any effect on gemcitabine resistance *in vivo*. Despite Hmga2 having no effect in gemcitabine-treated mouse models of pancreatic cancer, it remains possible that HMGA2 may still be a determinant of therapy sensitivity in human PDAC or of response to other chemotherapies.

We also investigated whether HMGA1, a HMGA2 family member with related functions, compensates for HMGA2 during PDAC metastasis. We and others have shown that HMGA1 and HMGA2 overlap in their expression in human PDAC and in *KP*^172^*CT* and *KP*^*Het*^*CT* mice (Fig. 4, ref.^32^). Additionally, the expression of HMGA1 and HMGA2 correlates with poor survival of PDAC patients^32^. Notably, PDAC patients with HMGA1^negative^ HMGA2^negative^ tumours have significantly better survival than all other patients (Fig. 4d). Cancer cells from *KP*^*Het*^*CT;Hmga2*^+/+^ and *KP*^*Het*^*CT;Hmga2*^*CK/CK*^ mice express Hmga1 at similar levels, therefore Hmga1 could compensate for Hmga2 deficiency to enable malignant transformation and metastasis. Knockdown of *Hmga1* in a PDAC cancer cell line generated from a *KP*^172^*CT;Hmga2*^*CK/CK*^ mouse had no impact on metastatic ability after intravenous transplantation. *Hmga1* knockdown in our system might not have been sufficient to entirely remove Hmga1 function, with remaining Hmga1 levels still being adequate to enable metastasis. Alternatively, a pro-metastatic effect of Hmga1/Hmga2 could be stable and not depend on continued Hmga1 expression. *In vivo* models that incorporate genetic deletion of both Hmga1 and Hmga2 will allow this question to be addressed definitively.

Taken together, we provide evidence that Hmga2 is functionally dispensable for the malignant transformation, progression, and metastatic ability of pancreatic cancer *in vivo*. Our results support Hmga2 being solely of prognostic value, as has been shown for multiple cancer types^29–31,34^. Hmga2 remains a prognostic marker which identifies an advanced cancer cell state in primary pancreatic tumours and marks a metastasis-driving subpopulation of cancer cells.

## Methods

### Mice

*Kras*^*LSL-G12D*^, *p53*^*LSL-R172H*^, *p53*^*flox*^, *Pdx1-Cre*, *Rosa26*^*LSL-tdTomato*^, and *Hmga2*^*CK*^ mice have been described^40,54–58^. For gemcitabine treatment, 28 day old *Kras*^*LSL-G12D/+*^;*p53*^*flox/flox*^;*R26*^*LSL-Tom/+*^;*Pdx1-Cre* and *Kras*^*LSL-G12D/+*^;*p53*^*flox/flox*^;*Hmga2*^*CK/CK*^;*R26*^*LSL-Tom/+*^;*Pdx1-Cre* were randomly separated into two groups each (mixed gender) and treated with four intraperitoneal injections of gemcitabine (120 mg/kg) or saline on day 28, 32, 35, and 39 of age. For transplantation of knockdown cell lines, 2 × 10^4^ cells were injected into the lateral tail vein of male 129/Bl6 F1 mice (Jackson Laboratories, Stock number 101043). Mice were analysed 3 weeks after injection. No statistical method was used to predetermine sample size. All experimental protocols were approved by the Stanford University Animal Care and Use Committee and performed in accordance with their guidelines.

### Histology and immunohistochemistry on mouse PDAC

Tissues were fixed in 4% formalin in PBS overnight and transferred to 70% ethanol prior to paraffin embedding. After de-parafinization, re-hydration, and antigen retrieval, IHC was performed on 4 μm sections with the ABC Vectastain kit (PK-4001, Vector Laboratories Inc.) with antibodies to Hmga2 (59170AP, BioCheck Inc.), Tomato (600-401-379, Rockland Inc.), GFP (ab6673, Abcam), CK19 (TROMA-III, Developmental Studies Hybridoma Bank), Insulin (A0564, Dako) and Hmga1 (sc-8982, Santa Cruz). Sections were developed with DAB and counterstained with haematoxylin. Haematoxylin and Eosin (H&E) staining was performed using standard methods.

### Tumour dissociation and cancer cell flow cytometric analysis

Pancreatic tumours and metastasis were dissociated into a single cell suspension prior to FACS analysis. Briefly, tumours were minced with sharp scissors and incubated for 20 minutes at 37^o^C in HBSS medium (Corning, 21-022-CV) with collagenase IV (Worthington, 1 mg/ml, LS004188) and dispase (Corning, 354235) followed by quenching with ice-cold L-15 medium (Invitrogen, 21083-027) containing 10% FBS and DNase (20 μg/mL). Cells were stained with Allophycocyanin (APC)-conjugated antibodies to CD45 (30-F11), CD31 (390), F4/80 (BM8), and Ter119 (TER-119, all from BioLegend) to exclude hematopoietic and endothelial cells. DAPI was used to exclude dead cells. Cells within the peritoneal cavity were collected immediately after euthanasia by making a small incision in the peritoneum followed by introduction of 1 ml PBS. For circulating tumour cell analysis blood was collected immediately after euthanasia by cardiac puncture. Red blood cell lysis was performed by incubation with ACK lysis buffer (155 mM NH_4_Cl, 10 mM KHCO_3_, 0.1 mM EDTA). BD LSR II analysers and FACSAria sorters (BD Biosciences) were used and FlowJo software was utilized for analysis.

### Immunohistochemistry for HMGA1 and HMGA2 on human PDAC

Expression of HMGA1 and HMGA2 was assessed using IHC on 4 μm sections of paraffin-embedded tissue from each TMA. Sections were heated at 60 °C for 60 minutes, de-paraffinized in xylene and rehydrated through a graded alcohol series. Three percent H_2_O_2_ block was used to quench endogenous peroxidase activity. Antigen retrieval was performed in a de-cloaking chamber using a Borg Decloaker RTU antigen retrieval solution (Biocare Medical). Sections were then blocked in normal horse serum (Vector Laboratories) and incubated in a humidified chamber overnight at 4 °C with a 1:1000 dilution of primary anti-HMGA1 antibody (sc-8982, Santa Cruz) or anti-HMGA2 antibody (59170AP, BioCheck). Slides were washed in PBS and incubated with biotinylated anti-rabbit secondary antibody (Vector Laboratories) for 30 minutes at room temperature. Staining was visualized using DAB (Vector Laboratories) at room temperature. Slides were then counterstained with haematoxylin, rinsed in water and dehydrated through a graded alcohol series and xylene, and mounted with VectaMount (Vector Laboratories). HMGA1 and HMGA2 staining of individual samples was evaluated manually and an arbitrary cutoff of staining intensity was used to determine if a sample was considered negative or positive. Overall survival (OS) was defined as the time of surgery to the date of death from any cause. OS was analysed using Kaplan–Meier and log-rank tests. Significance was determined as a p-value < 0.05.

This study was approved by the Stanford University Institutional Review Board and performed in accordance with their guidelines, with a waiver of informed consent to use patient tissue and specimens.

### Generation and selection of murine PDAC cell lines

We generated polyclonal cell lines from primary tumours and metastases that formed in the autochthonous *Kras*^*LSL-G12D/*+^*;Trp53*^*LSL-R*172*H/*+^*;Rosa*^*LSL-tdTomato*^*;Pdx1-Cre* pancreatic cancer mouse model with (*Hmga2*^+/+^) and without (*Hmga2*^*CK/CK*^) Hmga2 expression^22,54^. To establish the cell lines, a piece of the tumour or macro-metastasis was dissociated into a single cell suspension, washed twice with cold PBS, minced with a scalpel and transferred to a tissue culture dish containing DMEM media (high glucose with 10% FBS and antibiotics). Cells were allowed to attach and grown for one week with two media changes. Then cells were passaged at least 3 times to select away from fibroblast contamination. Purity was confirmed by FACS for tdTomato and MycoAlert Mycoplasma detection kit (Lonza) was used to verify the lack of Mycoplasma contamination.

### Lentiviral knockdown and qRT-PCR analysis

*Hmga1* and *Hmga2* were knocked down using pLKO lentiviral vectors; mouse sh*Hmga1*1 (TRCN0000182651) and mouse sh*Hmga*2 (TRCN0000126044). The control vector was pLKO-shLuciferase. Virus production, cell infection, and selection were performed as previously described^59^. *K*nockdown was confirmed by qPCR and western blotting. qRT-PCR for mouse *Hmga1* (Hmga1_Fw GGGGCAGACCCAAGAAAC and Hmga1_Rv GGCACTGCGAGTGGTGAT) and *Hmga2* (Hmga2_Fw GGATCCTGGCAGAAACTTCC and Hmga2_Rv AACGGGACAGAGATAGAGACTGA) was performed using standard SYBR green qPCR protocols and normalized to mouse *Gapdh* (*Gapdh*_Fw TTTGATGTTAGTGGGGTCTCG and *Gapdh*-Rv AGCTTGTCATCAACGGGAAG).

### Western blot analyses

Cells were pelleted before lysis with standard RIPA buffer supplemented with 1/100 volume of protease inhibitor cocktail (Sigma-Aldrich, P8340). Lysates were denatured in Laemmli buffer with β-mercaptoethanol before loading onto a precast 4–12% Bis-Tris PAGE gel (Invitrogen, NP0321BOX). Separated samples were then transferred onto a PVDF membrane (Bio-Rad, 162-0177) before staining with the following antibodies overnight: anti-Hmga2 (Biocheck, 59170AP, or Thermo Scientific, P52926), anti-Hsp90 (BD Biosciences, 610418), and anti-Hmga1 (Santa Cruz, sc-8982 or sc-393213). For the secondary antibodies, goat anti-rabbit IgG-HRP (Santa Cruz Biotech, sc-2004), goat anti-mouse IgG-HRP (Santa Cruz Biotech, sc-2005), and donkey anti-goat IgG-HRP (Santa Cruz Biotech, sc-2020) were used. For enhanced chemiluminescence, Thermo Scientific Pierce ECL 2 Western Blotting Substrate (PI80196) was used.

### Statistical analysis

Graphs and statistics were generated using GraphPad Prism software. Significance, where indicated, was calculated using the Wilcoxon test for non-normally distributed data. Survival was compared using Kaplan–Meier plots and log-rank tests. Significance was determined as a p-value < 0.05. No statistical method was used to predetermine sample size.

## Electronic supplementary material

Supplementary Figures 1-4

## Data Availability

No datasets were generated during the current study.
