# Supplementary material for: Hmga2 is dispensable for pancreatic cancer development, metastasis, and therapy resistance
Source: Sci Rep. 2018 Sep 18;8:14008. doi: 10.1038/s41598-018-32159-x (PMC6143627; doi:10.1038/s41598-018-32159-x)
Supplement: Supplementary file 1 — Supplementary Figures 1-4 [file 41598_2018_32159_MOESM1_ESM.pdf]

Hmga2 is dispensable for pancreatic cancer development, metastasis, and therapy resistance

Shin-Heng Chiou[1], Madeleine Dorsch[2], Eva Kusch[2], Santiago Naranjo[1], Margaret M. Kozak[3], Albert C. Koong[3,4], Monte M. Winslow[1,5,6*], and Barbara M. Grüner[2,7*]

Supplementary Information

Supplemental Figure S1

Supplemental Figure S2

Supplemental Figure S3

Supplemental Figure S4

Chiou *et al.* Supplemental Figure S1

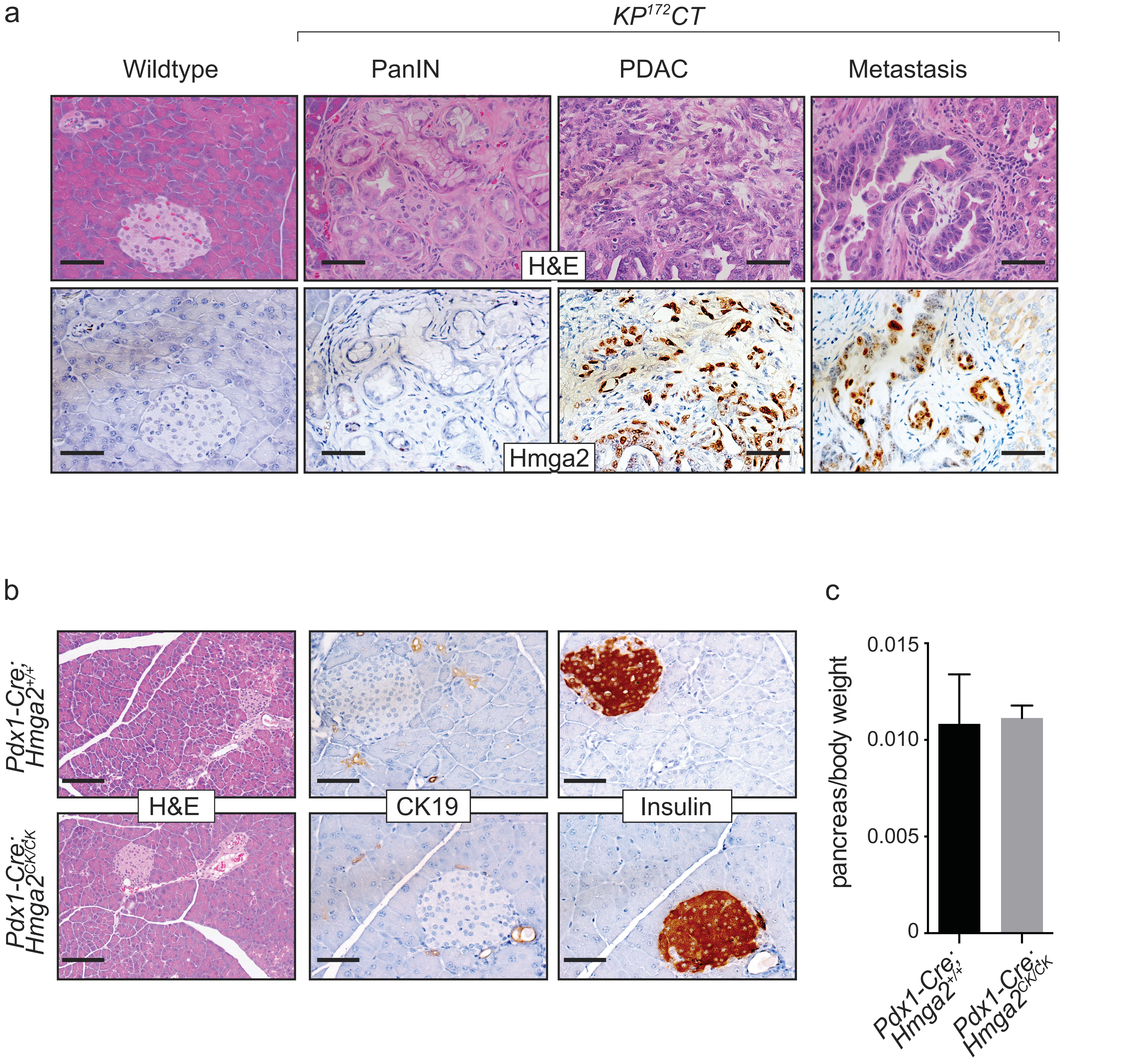

Supplemental Figure S1. Conditional inactivation of Hmga2 does not affect normal pancreas development.

a. H&E as well as IHC for Hmga2 of $KP^{172}CT$ and wild type mice shows that Hmga2 is expressed in pancreatic cancer and metastasis but not in normal tissue or precancerous lesions. Scale bars = 50 µm.

b. H&E and Immunohistochemical stainings for cytokeratin 19 (CK19) and insulin of mice with pancreas-specific Hmga2-inactivation (*Pdx1-Cre;*$Hmga2^{CK/CK}$) and wildtype (*Pdx1-Cre;*$Hmga2^{+/+}$) littermates at 4 weeks of age shows no difference in pancreas development and morphology. Scale bars H&E = 100 µm,
scale bars IHCs = 50 µm.

c. Pancreas to body weight ratios of *Pdx1-Cre;*$Hmga2^{CK/CK}$ and *Pdx1-Cre;*$Hmga2^{+/+}$ littermates at 11 weeks of age were not significantly different (n = 4 mice per group; $p = 0.3429$; mean +/- SEM is shown).

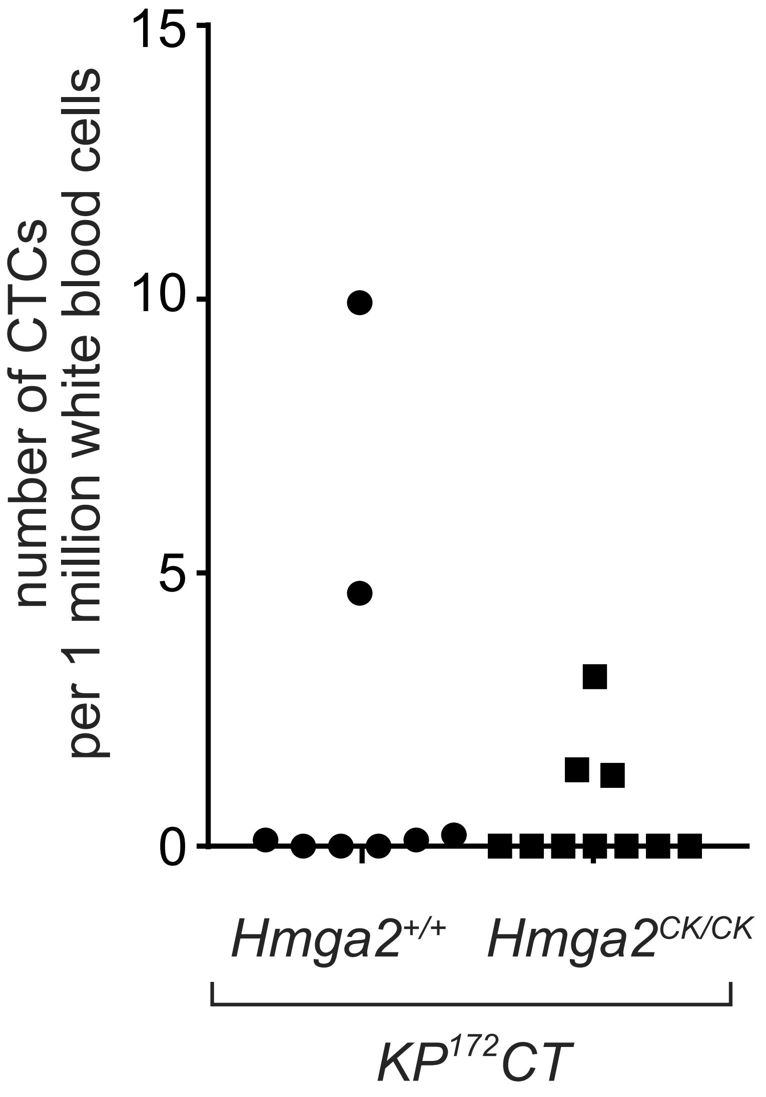

Supplemental Figure S2. Hmga2 deficiency does not alter the incidence and number of circulating tumour cells.

Number of circulating tumour cells (CTCs) per one million white blood cells is comparable but variable in *KP*^{172}*CT;Hmga2*$^{+/+}$ and *KP*^{172}*CT;Hmga2*$^{CK/CK}$ mice as detected by FACS. No significant differences in CTC number were observed.

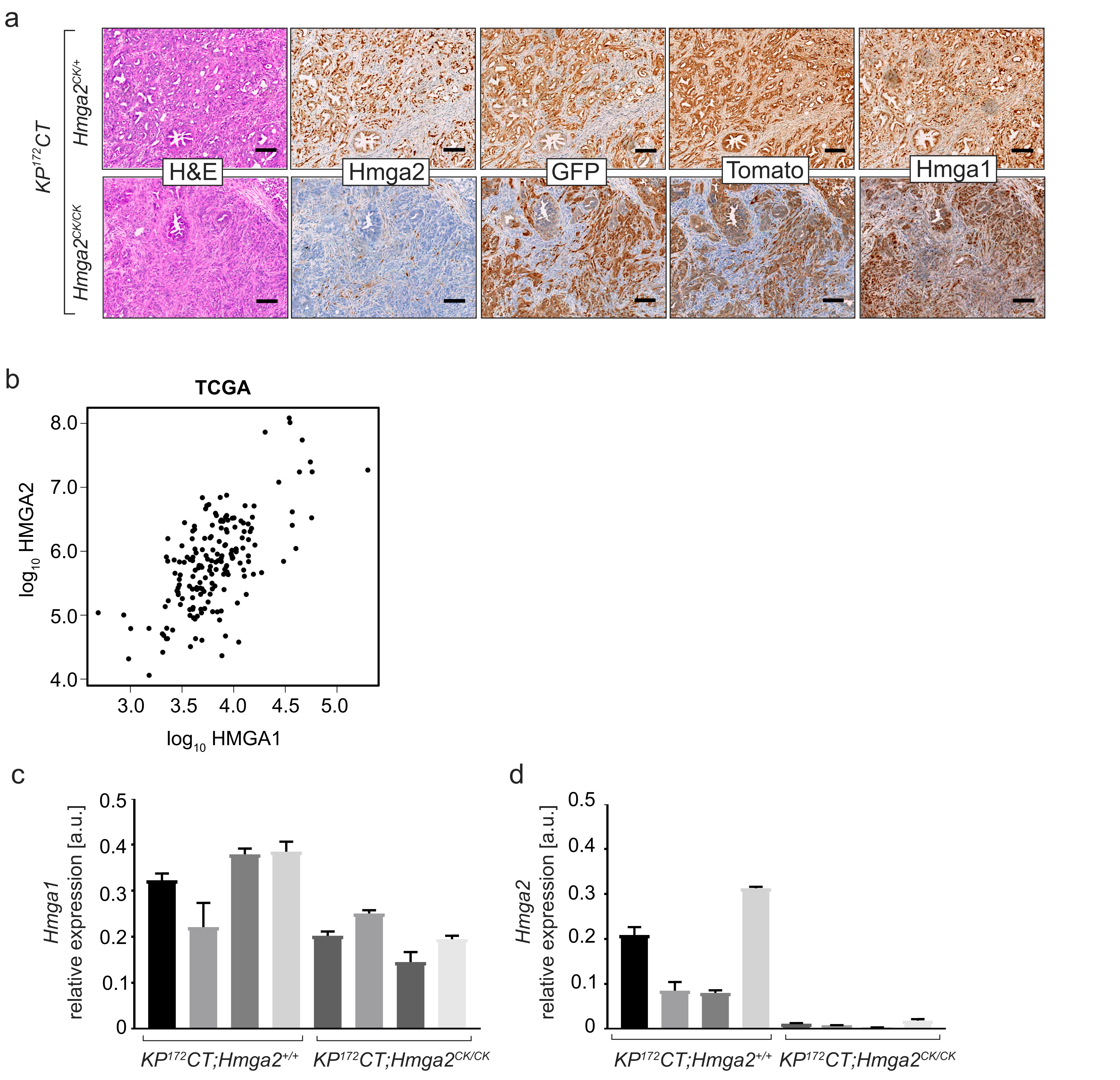

Supplemental Figure S3. Hmga1 and Hmga2 are co-expressed in pancreatic cancer.

a. Hmga2 expression in $KP^{172}CT;Hmga2^{CK/+}$ mice overlaps with expression of Hmga1 in GFP and Tomato positive cancer cells. Hmga2 is absent in pancreatic tumours from $KP^{172}CT;Hmga2^{CK/CK}$ mice but Hmga1 expression is unaffected. Scale bars = 100 µm.

b. TCGA dataset was interrogated for correlation of HMGA1 and HMGA2 expression. Each dot represents one sample. The correlation is 0.414 (calculated as Pearson correlation coefficient) or 0.581 (Spearman correlation coefficient), respectively.

c-d. qPCR for relative expression of *Hmga1* (**b**) and *Hmga2* (**c**) on cell lines isolated from pancreatic tumours from $KP^{172}C;Hmga2^{+/+}$ and $KP^{172}C;Hmga2^{CK/CK}$ mice (n = 4 cell lines per group, mean +/- SEM of triplicate wells is shown).

a

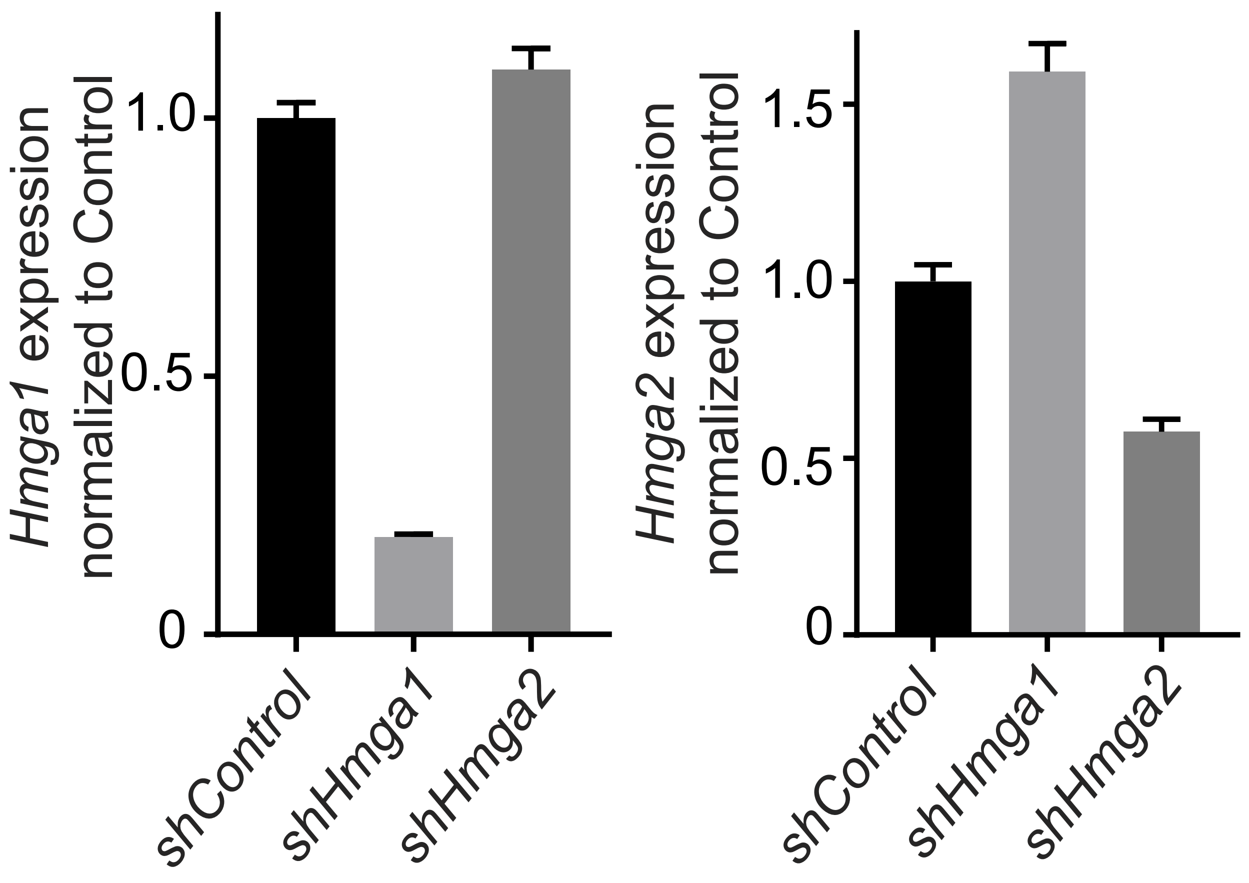

b

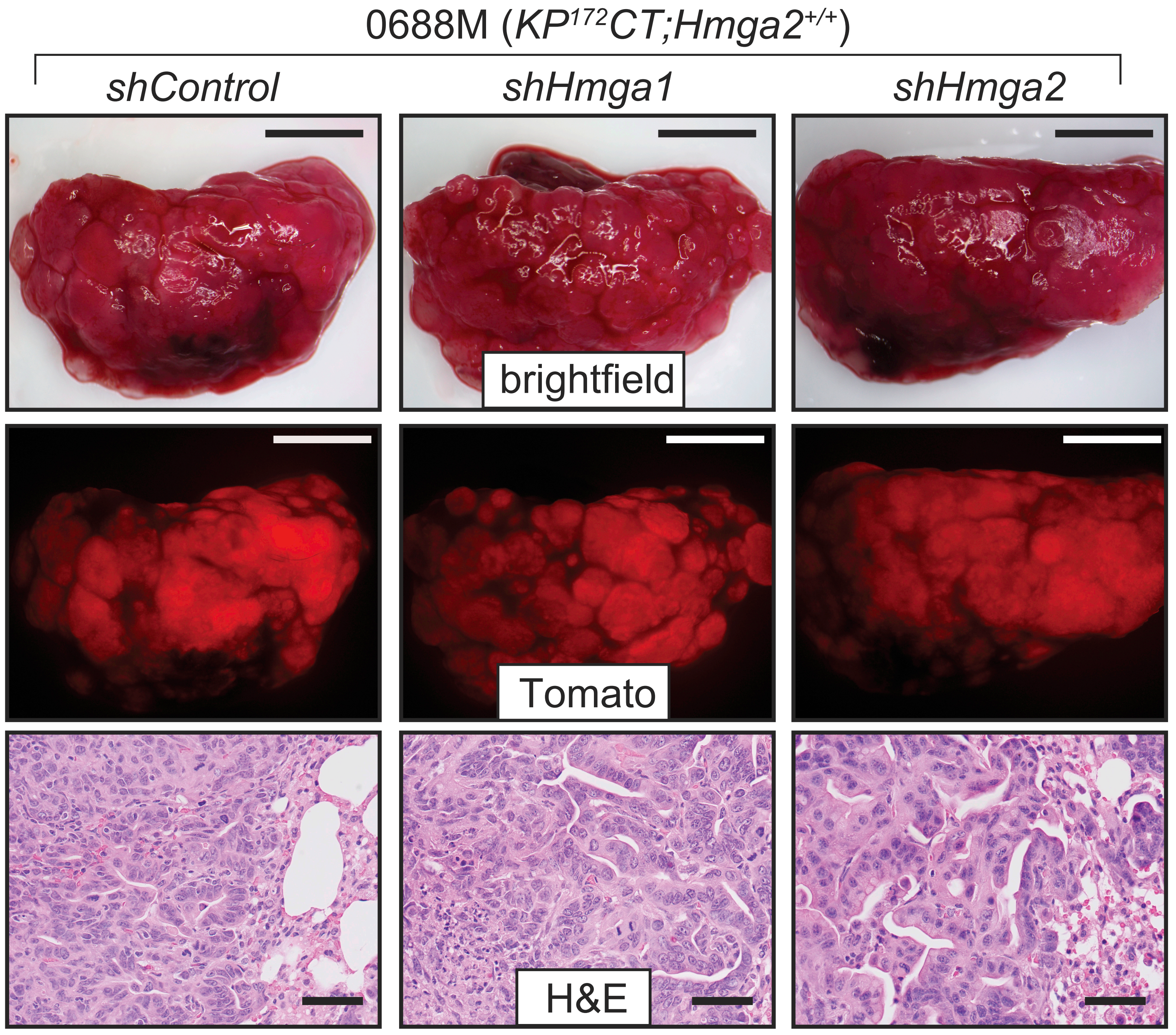

c

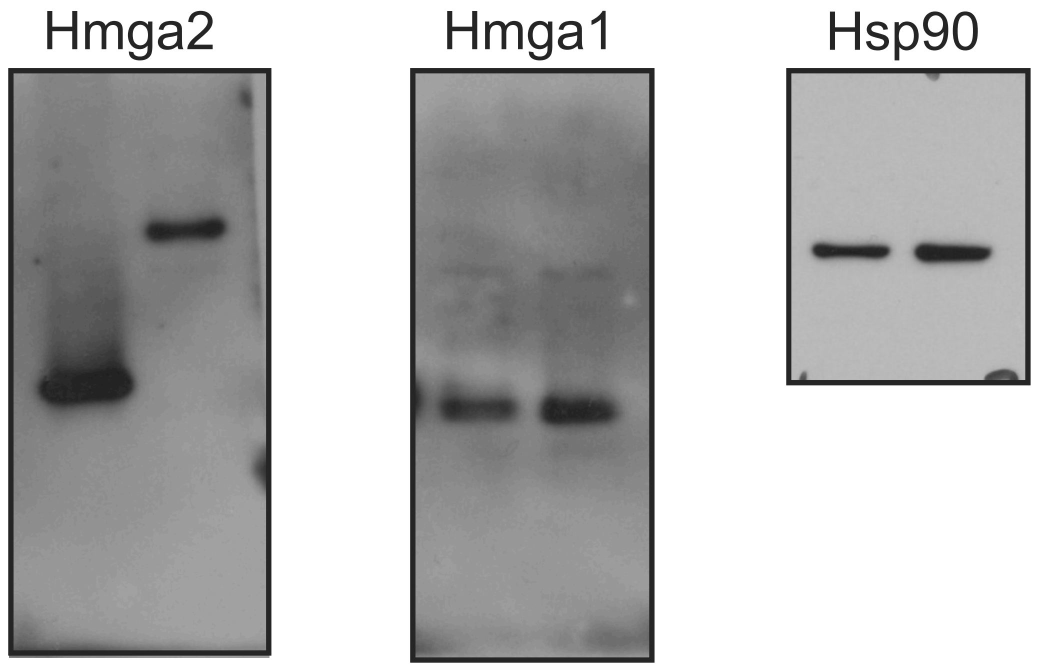

Supplemental Figure S4. Hmga1 and Hmga2 are co-expressed in pancreatic cancer.

a. shRNA-mediated knockdown of *Hmga1* or *Hmga2* in a cell line isolated from a liver metastasis in a *KP^{172}CT;Hmga2$^{+/+}$* mouse (0688M). Mean +/- SEM of triplicate wells is shown.

b. 0688M cells with shRNA-mediated knockdown of *Hmga1* or *Hmga2* showed no significant difference in metastatic potential upon intravenous injection into recipient mice in comparison to control cells (n = 3 mice per group, one representative image per group is shown). Scale bars upper and middle panel = 4 mm, scale bars lower panel = 50 µm.

c. Full-length blots corresponding to the cropped western blot images presented in main Figure 4b. For each probed protein, the same exposure was used for both samples. The samples were run twice on separate gels. One membrane was probed for Hmga1, the other was probed for Hmga2. Hsp90 was probed as loading control on the upper part (> 70 kDa) of both membranes, only one membrane is shown here, the other loading control depicted comparable results (data not shown).
